# Assessing Q Fever Exposure in Veterinary Professionals: A Study on Seroprevalence and Awareness in Portugal, 2024

**DOI:** 10.3390/vetsci12060512

**Published:** 2025-05-23

**Authors:** Guilherme Moreira, Mário Ribeiro, Miguel Martins, José Maria Cardoso, Fernando Esteves, Sofia Anastácio, Sofia Duarte, Helena Vala, Rita Cruz, João R. Mesquita

**Affiliations:** 1 ICBAS—School of Medicine and Biomedical Sciences, Porto University, 4050-313 Porto, Portugal; majojqr@gmail.com; 2 Centro de Investigação Vasco da Gama (CIVG)/Departamento de Ciências Veterinárias, Escola Universitária Vasco da Gama (EUVG), Campus Universitário, Av. José R. Sousa Fernandes, 3020-210 Coimbra, Portugal; migueljcmartins@gmail.com (M.M.); sofia.anastacio@gmail.com (S.A.); 3 José Maria Cardoso Ceva Saúde Animal, Rua Dr. António Loureiro Borges, 9/9A-9°A, Miraflores, 1495-131 Algés, Portugal; jose.cardoso@ceva.com; 4 Instituto Politécnico de Viseu, Escola Superior Agrária de Viseu, Campus Politécnico, 3504-510 Viseu, Portugal; festeves@esav.ipv.pt (F.E.); hvala@esav.ipv.pt (H.V.); rcpaiva@esav.ipv.pt (R.C.); 5 CERNAS-IPV Research Centre, Instituto Politécnico de Viseu, Campus Politécnico, Repeses, 3504-510 Viseu, Portugal; 6 Department of Animal Sciences and Veterinary, University Institute of Health Sciences—CESPU (IUCS-CESPU), 4585-116 Gandra, Portugal; 7 Center of Neurosciences and Cell Biology, Health Science Campus, 3000-548 Coimbra, Portugal; 8 LAQV—Associated Laboratory for Green Chemistry, REQUIMTE—Network of Chemistry and Technology, Laboratório de Bromatologia e Farmacognosia, Faculdade de Farmácia da Universidade de Coimbra, Polo III, Azinhaga de Santa Comba, 3000-548 Coimbra, Portugal; 9 Centre for the Research and Technology of Agroenvironmental and Biological Sciences, CITAB, Inov4Agro, Quinta de Prados, Edifício Reitoria, 5000-801 Vila Real, Portugal; 10 EpiUnit—Instituto de Saúde Pública da Universidade do Porto, Laboratory for Integrative and Translational Research in Population Health (ITR), Rua das Taipas, nº 135, 4050-091 Porto, Portugal; 11 Centro de Estudos de Ciência Animal (CECA), Instituto de Ciências, Tecnologias e Agroambiente (ICETA), Universidade do Porto (UP), Rua D. Manuel II, Apartado 55142, 4051-401 Porto, Portugal; 12 Associate Laboratory for Animal and Veterinary Science (AL4AnimalS), 1300-477 Lisboa, Portugal

**Keywords:** *Coxiella burnetii*, occupational exposure, Q fever, epidemiology, coxiellosis

## Abstract

Due to their frequent contact with animals, veterinarians may be at preferential risk of *Coxiella burnetii* exposure due to occupational contact with livestock. This study assesses the seroprevalence and risk factors associated with *C. burnetii* seropositivity in Portuguese veterinarians. A cross-sectional study compared IgG anti-*C. burnetii* in veterinarians’ sera to a demographically matched control group. Univariate and multivariate logistic regression analyses evaluated associations between the demographic, occupational, and biosecurity factors and seropositivity. Seroprevalence among veterinarians was 33.7%, significantly higher (*p* = 0.0023) than in the controls (17.39%). Univariate analysis identified higher seropositivity in the northern region (*p* = 0.03), though this association was not significant after adjustment (*p* = 0.07). Protective measures, including isolating aborting animals from the rest of the herd (adjusted OR [aOR]: 0.35, *p* = 0.03) and wearing gloves during sample collection (OR: 0.28, *p* = 0.009), were significantly associated with lower infection risk. Veterinarians face increased *C. burnetii* exposure, but specific biosecurity practices reduce risk. Strengthening preventive measures, including personal protective equipment (PPE) use and biosecurity training, is essential to mitigate occupational and public health risks. Further research should explore vaccination strategies and molecular epidemiology to improve risk reduction efforts.

## Simple Summary

Veterinarians have a higher risk of *Coxiella burnetii* exposure due to contact with livestock. This study examines its prevalence and risk factors in Portuguese veterinarians. *C. burnetii* antibody titers in veterinarians and in a matched control group were compared. Logistic regression analyzed demographic, occupational, and biosecurity factors. The seroprevalence was 33.7% in veterinarians, which was higher (*p* = 0.0023) than that in the controls (17.4%). Northern veterinarians had higher seropositivity (*p* = 0.03), but this was not significant after adjustment (*p* = 0.07). Isolating aborting animals from the rest of the herd (OR: 0.35, *p* = 0.03) and wearing gloves during sampling (OR: 0.28, *p* = 0.009) reduced the risk. Veterinarians have higher *C. burnetii* exposure rates, but proper biosecurity lowers this risk. Personal protective equipment (PPE) use and training should be reinforced. Further research on the vaccination and epidemiology of this agent is needed.

## 1. Introduction

*Coxiella burnetii* is an obligate intracellular, Gram-negative coccobacillus and the causative agent of Q fever, a zoonotic disease that presents a wide range of clinical manifestations in humans and animals [1]. Horizontal transmission primarily occurs via inhalation of aerosolized particles contaminated with the bacterium’s environmentally resistant small cell variant, often from parturient fluids or other excreta of infected animals [2]. Direct contact and consumption of unpasteurized dairy products are less common routes, while tick bites, though relatively infrequent, represent a recognized mode of transmission [3].

In arthropod vectors, particularly ticks, *C. burnetii* populations are maintained through both transstadial and transovarial transmission. These vertical transmission pathways contribute to the persistence of the pathogen in natural cycles and may play a role in both animal and human infections [1,3,4].

Q fever in humans can present as either an acute or chronic illness. Acute Q fever most commonly manifests as a non-specific flu-like syndrome, atypical pneumonia, or hepatitis. Rarely, more severe complications such as acute endocarditis or meningoencephalitis may occur. Chronic Q fever typically develops in individuals with underlying risk factors such as immunosuppression, prosthetic implants, or preexisting valvular heart disease. It is most frequently associated with infective endocarditis and vascular infections, including infected aneurysms and vascular grafts. Less commonly, it may involve pulmonary, hepatic, or osteoarticular sites [5,6]. In pregnant women, *C. burnetii* infection may lead to obstetric complications such as miscarriage, premature delivery, or fetal growth restriction. Despite its pathogenic potential, *C. burnetii* infections are frequently asymptomatic, particularly in healthy individuals and in a variety of domestic and wild animal reservoirs [7,8,9].

According to [1,2,3,4,5,6,7,8,9], ruminants, particularly small ruminants such as sheep and goats, are considered primary reservoirs of *C. burnetii* [10]. Infected animals often do not exhibit clinical signs, but when they do, reproductive disorders, including abortions, stillbirths, weak offspring, mastitis, metritis, and infertility are the most frequently associated signs. Infected animals shed *C. burnetii* in their feces, urine, milk, and particularly in birth products, with abortive tissues such as the placenta and amniotic fluid containing markedly higher bacterial loads [11]. This shedding significantly contributes to environmental contamination and the ongoing transmission cycle of the pathogen. Apart from domestic animals, wildlife also plays a role in the epidemiology of Q fever. Rodents, deer, and other mammals can harbor *C. burnetii*, serving as additional sources of infection for humans and livestock [12,13,14].

Humans become infected primarily by inhaling contaminated aerosols or dust particles from environments contaminated with *C. burnetii*. This mode of transmission makes occupational exposure a significant risk factor. Individuals who frequently work with animals, particularly small ruminants, are at an elevated risk of infection. High-risk groups include veterinarians [15], farmers, livestock handlers, abattoir and slaughterhouse workers, laboratory personnel handling infected samples, and individuals living in rural areas with high levels of livestock farming [15,16,17,18]. Additionally, foresters and those involved in wildlife management are also susceptible due to potential contact with infected wild animal populations. Infections can also occur through the consumption of unpasteurized dairy products from infected animals, though this is a less common route [19].

One of the most concerning aspects of *C. burnetii* is its exceptional resistance to environmental influences. The bacterium can survive desiccation, high temperatures, and exposure to common disinfectants, which contributes to its persistence in the environment. In its spore-like form, *C. burnetii* can remain viable for extended periods—up to 10 months on sheep wool and up to 40 months in powdered milk stored at room temperature. This remarkable resilience underscores the importance of stringent biosecurity measures to prevent the spread of Q fever, particularly in livestock farming and veterinary practices [20].

Coxiellosis poses a significant threat to both public and animal health, with economic repercussions due to reproductive losses and decreased productivity in affected livestock. The disease can lead to increased culling rates, reduced milk production, and higher veterinary costs, making it a considerable concern for the agricultural sector and food industry. Effective surveillance, vaccination, and management strategies are crucial for controlling the spread of *C. burnetii* and minimizing its impact on animal and human populations [21,22].

Vaccination remains a cornerstone in *C. burnetii* infection control by significantly reducing the incidence of reproductive losses and minimizing environmental contamination, thereby limiting onward transmission to susceptible animals and humans. Currently, two veterinary vaccines are commercially available in various regions worldwide. Coxevac^®^ (CEVA Santé Animale, Libourne, France) is a non-adjuvanted, inactivated vaccine formulated with phase I *C. burnetii* (Nine Mile strain) which is licensed for use in goats and cattle. Chlamyvax^®^ (Mérial, Lyon, France) is a bivalent inactivated vaccine composed of Chlamydia abortus and phase II *C. burnetii* which is intended for immunization of sheep and goats [21].

Despite its relevance as an occupational zoonosis, Q fever remains an understudied disease in many regions, including Portugal. In contrast to the prevalence of *C. burnetii* in livestock populations [23,24], limited research has been conducted on the awareness and seroprevalence of *C. burnetti* among veterinarians who work closely with farm animals. This gap in knowledge is particularly concerning, given the high-risk nature of veterinary work, which involves regular exposure to potentially infected animals and contaminated environments.

The objective of this study is to address this knowledge gap by evaluating the awareness levels of Portuguese farm animal veterinarians regarding Q fever as well as assessing their perceived risk of exposure and past infection history. By conducting a comprehensive survey and serological testing among veterinarians in Portugal, this study aims to determine the prevalence of Q fever antibodies in this professional group, shedding light on the extent of occupational exposure and informing future public health strategies. Ultimately, the findings from this research could contribute to improved preventive measures, including vaccination campaigns, enhanced biosecurity protocols, and greater educational outreach efforts targeting veterinary professionals.

Given the growing recognition of Q fever as a major public health concern worldwide [21,22], it is essential to improve surveillance and prevention strategies to mitigate its impact. Veterinary professionals play a crucial role in controlling the spread of *C. burnetii*, not only by diagnosing and managing infections in livestock but also by implementing measures to protect themselves and their colleagues. By increasing awareness and promoting proactive risk management, the veterinary community can help reduce the burden of Q fever and safeguard both animal and human health.

## 2. Materials and Methods

This cross-sectional study was designed to assess the seroprevalence of infection in two distinct human population cohorts.

**Figure 1:**
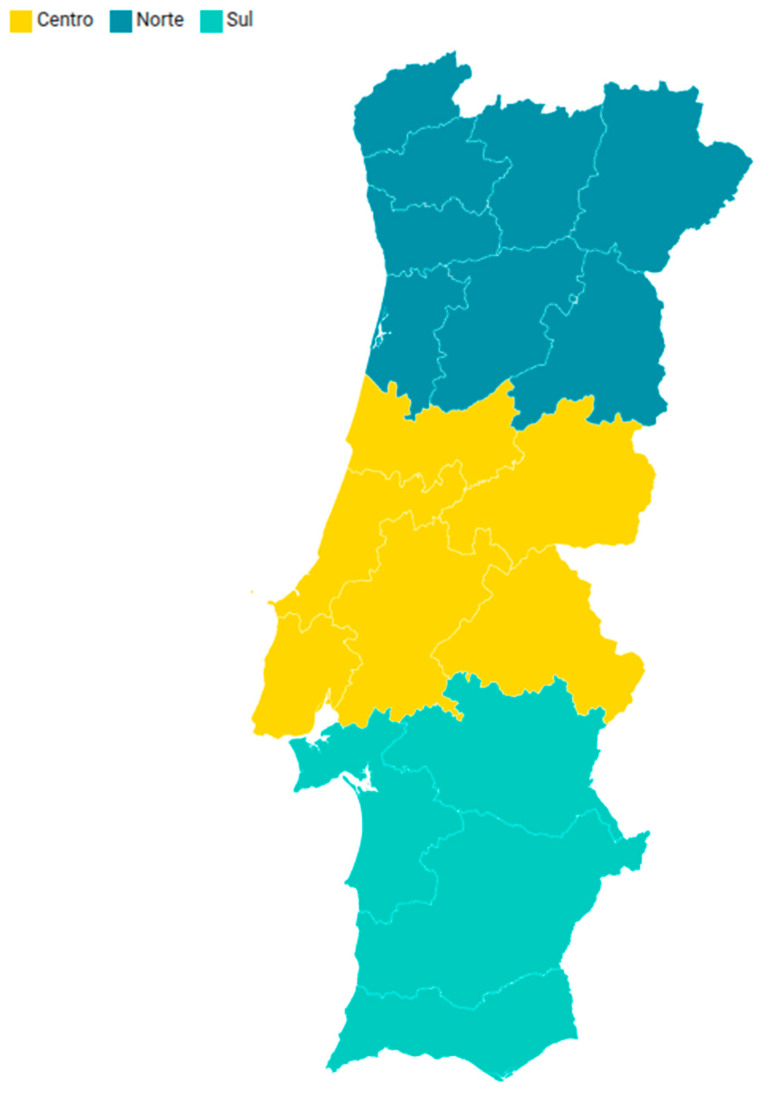
Geographical division of Portugal into three macro-regions: *Norte* (North), *Centro* (Centre), and *Sul* (South).

For the study group, participants were selected through a convenience sampling method during scientific seminars focused on Q fever which were specifically organized for veterinarians. These seminars were held across mainland Portugal between February and March 2024 and targeted active members of the Portuguese Veterinary Medical Association (OMV) who provide veterinary services to livestock farms. Informed consent was obtained from all participants after a comprehensive explanation of the objectives and procedures of the study. Additionally, a structured questionnaire was administered to collect sociodemographic and occupational information. The questionnaire was divided into two sections. The first section aimed to gather sociodemographic data on the veterinarians, including the main geographic region of professional activity (Figure 1), while the second focused on disease-related aspects, including biosecurity practices on farms, animal handling methods during peripartum procedures, the use of personal PPE, diagnostic practices, and knowledge of the epidemiology of Q fever and of previous confirmed cases in both farmers and veterinarians.

For the control group, sera from blood donors with no direct or indirect contact with farm animals or agricultural environments that could expose them to *C. burnetii* or other zoonotic pathogens associated with farm animals were used. These samples were obtained from an unrelated research project, and their utilization in studies investigating zoonotic agents was approved by the Ethics Committee of EUVG (CE-EUVG decision nº 01/2024). The control sera were selected at a 1:2 ratio, meaning two control samples were matched with each participant from the study group. Matching was performed based on age group, geographical location, and gender, ensuring demographic comparability between the study and control groups.

This study involved the analysis of a total of 276 serum samples, of which 92 samples corresponded to the study group and 184 corresponded to the control group.

Blood samples were collected by nurses with active registration within the respective professional associations, following good practice guidelines and other applicable regulations. The samples were collected in tubes containing a coagulation activator using a tube holder and 23Gx1 ½″ needles, all of which were part of the Vacumed^®^ (FL Medical S.R.L., Torreglia, Italy) system. The samples were refrigerated at 4 °C during transport to the laboratory. The samples were centrifuged at 3000 rpm for 15 min (ZIP-IQ^®^ centrifuge, LW Scientific, Lawrenceville, GA, USA). The resulting serum was transferred to sterile and properly labeled 2 mL microtubes. After this process, the serum samples were immediately frozen at −20 °C, and this temperature was maintained until the laboratory analysis was performed. For the control group, aliquots of serum samples previously stored at −20 °C were thawed.

The sera were tested for the presence of anti-*C. burnetii* IgG antibodies using an indirect commercial enzyme-linked immunosorbent assay (ELISA) test, *COXIELLA* BURNETII ELISA FASE II IgG^®^ (Vircell Microbiologists, Granada, Spain), according to the instructions of the manufacturer. The sensitivity and specificity of this assay were reported to be 95% and 97%, respectively (Vircell Microbiologists, as per the internal validation report of the manufacturer). Manufacturer-provided positive and negative controls were included in each run. Samples with an index lower than 9 were considered negative, samples with an index equal to or between 9 and 11 were considered equivocal, and samples with indexes greater than 11 were considered positive. Equivocal samples were retested, and if still equivocal, then they were considered negative. Exact 95% binomial confidence intervals (CIs) were calculated for proportions.

Data processing was performed using Excel^®^ software and organized into three sections according to the structure of the questionnaire. Responses to the questions were categorized, and in the absence of a response, the blank space was considered as a missing value during data processing. The categorization was based on veterinarians’ sociodemographic information, biosecurity practices on farms, animal handling methods during peripartum procedures, the use of personal protective equipment (PPE), diagnostic practices, and knowledge of Q fever epidemiology, including confirmed cases among both farmers and veterinarians.

Statistical analysis and data processing were performed using a combination of Microsoft Excel (Version 2410, Build 18129.20116), R (version 4.2.3), and custom Python (version 3.12.10) scripts. Only values with *p* < 0.05 were considered statistically significant. Values with *p* < 0.1 were described as borderline significant.

## 3. Results

Within the veterinarians group (*n* = 92), 31 individuals (33.7%) tested seropositive, whereas 61 (66.3%) were seronegative. In the control group (*n* = 184), seropositivity was detected in 32 individuals (17.39%), with the remaining 152 (82.61%) testing negative (Table 1).

Statistical analysis revealed a strong association between veterinary occupation and seropositivity. The calculated odds ratio (OR) of 2.41 (95% CI: 1.37–4.26) indicates that veterinarians had more than twice the odds of exhibiting serological evidence of previous *C. burnetii* exposure compared with the control group. The chi-square test for independence yielded a statistically significant result (X^2^ = 9.256, df = 1, *p* = 0.0023), confirming a non-random distribution of seropositivity across groups and confirming that the seroprevalence of *C burnetii* IgG antibodies was significantly higher among veterinarians compared with the control population.

**Figure 2:**
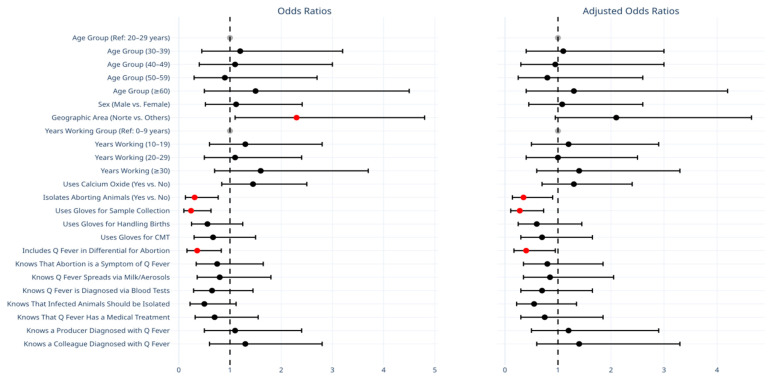
Comparison of odds ratios (ORs) and adjusted odds ratios (AORs) for demographic, occupational, and knowledge-related variables associated with *C. burnetii* seropositivity. Black circles represent unadjusted OR, while red circles indicate AOR after adjusting for counfounding variables. Error bars denote 95% confidence intervals. Reference categories are depicted as greyed-out markers positioned at the null value (e.g., OR = 1 or log OR = 0) and do not display confidence intervals.

A univariate logistic regression analysis was conducted to evaluate the association between demographic, occupational, and knowledge-related variables and seropositivity for *C. burnetii*. The results are summarized in Figure 2 and Table 2.

Geographic location was significantly associated with seropositivity, with individuals from the Northern region exhibiting higher odds of testing positive compared with other regions (OR: 2.30, 95% CI: 1.10–4.80, *p* = 0.03). Certain protective behaviors were also significantly associated with reduced odds of seropositivity. Specifically, individuals who isolated aborting animals had significantly lower odds of testing positive (OR: 0.31, 95% CI: 0.13–0.77, *p* = 0.01). Similarly, wearing gloves during sample collection was associated with a significantly lower likelihood of *C. burnetii* seropositivity (OR: 0.24, 95% CI: 0.10–0.63, *p* = 0.003). Including Q fever in the differential diagnosis of abortion cases was also associated with lower seropositivity (OR: 0.36, 95% CI: 0.16–0.83, *p* = 0.02). Conversely, the variables of age, gender, years of experience, and knowledge of Q fever transmission were not significantly associated with seropositivity.

A multivariate logistic regression model was constructed to adjust for potential confounders (Table 2). After adjusting for covariates, the association between geographic location and seropositivity was no longer statistically significant (adjusted OR: 2.10, 95% CI: 0.95–4.65, *p* = 0.07). However, the protective effect of isolating aborting animals remained significant (adjusted OR: 0.35, 95% CI: 0.14–0.90, *p* = 0.03). Wearing gloves during sample collection also continued to be significantly associated with lower odds of seropositivity (adjusted OR: 0.28, 95% CI: 0.11–0.73, *p* = 0.009). Including Q fever in the differential diagnosis for abortion cases retained their protective effect (adjusted OR: 0.40, 95% CI: 0.17–0.95, *p* = 0.04). Other variables, including years of experience, gender, and glove use for other tasks, did not show statistically significant associations.

## 4. Discussion

This study provides robust evidence of a higher seroprevalence of *C. burnetii* antibodies among veterinarians compared with a demographically matched control population. The overall seropositivity rate in the study group was 33.7%, which is significantly higher than the 17.39% observed in the control group. Statistical analysis demonstrated a strong association between the veterinary profession and prior exposure to *C. burnetii*, with an odds ratio (OR) of 2.41 (95% CI: 1.37–4.26, *p* = 0.0023), indicating that veterinarians were more than twice as likely to exhibit serological evidence of infection.

Both univariate and multivariate analyses highlighted key factors influencing *C. burnetii* seropositivity. Geographic location was initially associated with higher seropositivity, particularly in the Northern region (*p* = 0.03), where more intensive and clustered livestock production systems are common compared with the typically smaller-scale, extensive systems of the south, but this association lost significance after adjusting for confounders (adjusted OR: 2.10, 95% CI: 0.95–4.65, *p* = 0.07). In contrast, specific biosecurity measures consistently showed a protective effect. Isolating aborting animals (adjusted OR: 0.35, 95% CI: 0.14–0.90, *p* = 0.03) and using gloves during sample collection (adjusted OR: 0.28, 95% CI: 0.11–0.73, *p* = 0.009) were significantly associated with lower seropositivity, emphasizing the importance of these practices in reducing occupational exposure. These findings reinforce the need for targeted interventions to mitigate the risk of *C. burnetii* infection among veterinarians.

Our findings align with prior research demonstrating an elevated prevalence of *C. burnetii* exposure among individuals with occupational contact with livestock [17,25,26,27]. Studies conducted in various regions have consistently reported that veterinarians [26,27,28], farmers [29,30,31], abattoir workers [17], and laboratory personnel working with animal specimens [15] face a heightened risk of infection. Although reports from the Netherlands, Australia, and France have documented similar seroprevalence rates [32], variations in prevalence also exist across different studies, influenced by factors such as geographic location, livestock density, climate, biosecurity measures, and study design [33,34,35].

Our results indicate that isolating aborting animals and using gloves during sample collection had a protective effect against *C. burnetii* infection. The practice of isolating aborting animals might substantially reduce the risk of Q fever in veterinarians by limiting exposure to highly infectious birth materials, which are a major source of *C. burnetii*-containing aerosols. Likewise, the use of gloves during sample collection may help prevent direct contact with contaminated fluids and tissues, thereby decreasing the likelihood of infection. Together, these measures might play an important role in minimizing occupational exposure and may contribute to lower seropositivity rates among veterinarians.

Compared with studies conducted in regions with known Q fever outbreaks, the observed seroprevalence in the current study was relatively low [36,37,38]. This may reflect differences in exposure intensity, the effectiveness of biosecurity protocols, or the presence of vaccination programs for high-risk groups. In contrast, studies from endemic areas—such as the large Q fever outbreak in the Netherlands (2007–2010)—have reported significantly higher seroprevalence rates in exposed populations [30,33,38]. Such differences suggest that local epidemiological factors, including environmental persistence of *C. burnetii*, host–pathogen interactions, and public health interventions, play a crucial role in determining infection risk [39,40,41].

Currently, there is no human vaccine for Q fever authorized for routine use in Europe. The only licensed human vaccine, Q-Vax^®^—an inactivated whole-cell vaccine derived from the Henzerling phase I strain—is approved exclusively in Australia for high-risk populations. Its broader adoption has been limited due to concerns about adverse reactions and the requirement for pre-vaccination screening, including serological and skin tests [22].

Another important point of comparison is the relationship between seropositivity and clinical manifestations of Q fever. While some reports have identified strong correlations between seroprevalence and symptomatic individuals, others—including findings in the current study—suggest that exposure associated with asymptomatic or subclinical disease manifestations is common [42,43]. This highlights the potential for underdiagnosis, as individuals may acquire a *C. burnetii* infection without developing noticeable clinical signs, thereby remaining undetected in clinical settings. A study from Germany, for example, found that a substantial proportion of seropositive individuals had no recollection of experiencing Q fever-related symptoms [44], reinforcing the need for targeted screening in high-risk populations.

Molecular epidemiology studies conducted in various settings have further revealed strain-specific differences in virulence, transmission dynamics, and host preference, which may contribute to the observed variations in seroprevalence [45,46,47]. For instance, research utilizing whole-genome sequencing of *C. burnetii* strains has demonstrated genetic diversity among isolates from different hosts and geographic regions [48]. Understanding these genetic differences could provide insights into the potential for zoonotic spillover and inform control strategies. Expanding the present study to include molecular characterization of circulating strains in both human and animal populations would enhance the understanding of *C. burnetii* epidemiology and improve risk assessment strategies [49,50,51].

Frequent and direct contact with livestock, particularly during peripartum events, is a key risk factor for *C. burnetii* exposure. This organism is highly infectious and can be transmitted via aerosolized particles from birth products, urine, feces, and milk [28,29,41]. Inconsistent adherence to biosecurity measures in high-risk scenarios may further elevate exposure levels.

This study highlights the protective effect of specific biosecurity measures, particularly the use of gloves during sample collection and the isolation of aborting animals, both of which were associated with significantly lower odds of seropositivity. However, the persistently high seroprevalence suggests gaps in implementation, influenced by factors such as limited PPE access, variability in compliance, and time constraints during emergency interventions. Addressing these barriers is essential, as even minor lapses in protective measures can increase occupational exposure. Aditionally, considering Q fever as a potential cause of abortion seemed to protect veterinarians from *C. burnetii* infection, suggesting that increased awareness leads to better protective measures [15,50,52].

From a public health perspective, the elevated seroprevalence among veterinarians raises concerns about potential transmission risks, particularly for professionals working closely with infected animals or in contaminated environments. Veterinarians play a critical role in zoonotic disease surveillance and control, and their awareness of Q fever as a differential diagnosis for abortion cases is vital for early detection and intervention. Our findings suggest that routinely considering *C. burnetii* in diagnostic evaluations may reduce exposure risk by facilitating timely case identification and management.

The environmental resilience of *C. burnetii* presents additional challenges for farm-level biosecurity. This bacterium can persist for extended periods in dust, soil, and animal housing, leading to ongoing transmission risks [20,22]. Stricter sanitation protocols, proper disposal of birth materials, and quarantine measures for infected animals may help mitigate exposure. Additionally, exploring vaccination as a preventive strategy, particularly for veterinarians and livestock workers in endemic regions, could further reduce infection risk [49,53,54].

Overall, these findings emphasize the necessity of a multi-faceted approach to mitigating both occupational and public health risks associated with *C. burnetii*. Strengthening educational programs, reinforcing PPE usage, and improving adherence to farm-level biosecurity protocols are key strategies for reducing exposure among veterinarians. Furthermore, enhanced surveillance efforts and improved risk communication between veterinary professionals, farmers, and public health authorities will be critical in limiting zoonotic transmission and improving disease control efforts [16,18,25,44,55].

This study has several limitations. For one, the use of convenience sampling for veterinarians may have introduced selection bias, potentially limiting the generalizability of our findings. Additionally, seroprevalence data reflect past exposure rather than active infection, making it difficult to determine the timing or source of exposure. The control group, while matched by demographic factors, may not fully account for unmeasured confounders influencing seropositivity.

Future research should focus on several key areas to enhance our understanding of *C.* exposure in occupational settings. Longitudinal studies assessing seroconversion over time would provide valuable insight into infection dynamics among veterinarians and other high-risk groups, helping to identify early markers of infection and evaluate preventive measures.

Further investigation into the effectiveness of targeted biosecurity interventions, such as improved PPE, enhanced disinfection protocols, and vaccination strategies, is essential. These studies could optimize existing safety measures and establish evidence-based guidelines to reduce exposure risk in veterinary settings.

Expanding research to include other high-risk occupational groups, such as farmers, livestock handlers, and public health workers, would provide a broader perspective on risk factors and seroprevalence across professions. Comparing data across multiple high-risk groups could help identify common exposure pathways and inform comprehensive public health strategies.

Finally, conducting molecular epidemiology studies to characterize the genetic diversity of *C. burnetii* strains in both human and animal populations is of the utmost importance. Understanding transmission pathways, strain variations, and zoonotic risks could enhance diagnostic and preventive efforts, leading to more targeted interventions and a reduced burden of Q fever.

## 5. Conclusions

This study underscores the occupational risk veterinarians face regarding exposure to *C. burnetii*. The significantly higher seroprevalence in veterinarians highlights the need for enhanced biosecurity measures, particularly during sample collection and peripartum handling. Preventive strategies such as isolating aborting animals, using gloves, and incorporating Q fever into differential diagnoses for abortion cases can significantly reduce exposure risk. These findings emphasize the importance of targeted training, improved biosecurity protocols, and increased awareness to mitigate both occupational and public health risks associated with *C. burnetii*.

## Figures and Tables

**Table 1:** Seropositivity results for IgG anti-*C. burnetii* in veterinarians and controls.

Group	Positive Cases Absolute Frequency (*n*)	Positive Cases Relative Frequency (%)	Negative Cases Absolute Frequency (*n*)	Negative Cases Relative Frequency (%)	Total (*n*)
Test Group	31	33.70%	61	66.30%	92
Control Group	32	17.39%	152	82.61%	184
Total					276

**Table 2:** Univariate and multivariate risk factor analysis. Statistically significant values (*p* < 0.05) are shown in bold.

Variable	OR (95% CI)	*p* Value	Adjusted OR (95% CI)	*p* Value
Age Group				-
20–29 Years	1.00 (Ref)	-	1.00 (Ref)	
30–39 Years	1.20 (0.45–3.20)	0.72	1.10 (0.40–3.00)	0.86
40–49 Years	1.10 (0.40–3.00)	0.85	0.95 (0.30–3.00)	0.93
50–59 Years	0.90 (0.30–2.70)	0.86	0.80 (0.25–2.60)	0.71
≥60 Years	1.50 (0.50–4.50)	0.47	1.30 (0.40–4.20)	0.66
Gender (Male vs. Female)	1.12 (0.52–2.41)	0.72	1.08 (0.45–2.60)	0.86
Geographic Area (Norte vs. Others)	2.30 (1.10–4.80)	**0.03**	2.10 (0.95–4.65)	0.07
Years Working Group (Ref: 0–9 Years)	1.00 (Ref)	-	1.00 (Ref)	-
- 10–19 Years	1.30 (0.60–2.80)	0.51	1.20 (0.50–2.90)	0.68
- 20–29 Years	1.10 (0.50–2.40)	0.83	1.00 (0.40–2.50)	0.99
- ≥30 Years	1.60 (0.70–3.70)	0.28	1.40 (0.60–3.30)	0.45
Uses Calcium Oxide **(Yes vs. No)**	1.45 (0.84–2.50)	0.18	1.30 (0.70–2.40)	0.41
Isolates Aborting Animals (Yes vs. No)	0.31 (0.13–0.77)	**0.01**	0.35 (0.14–0.90)	**0.03**
Uses Gloves for Sample Collection	0.24 (0.10–0.63)	**0.003**	0.28 (0.11–0.73)	**0.009**
Uses Gloves for Handling Parturitions	0.56 (0.25–1.25)	0.15	0.60 (0.25–1.45)	0.26
Uses Gloves for CMT	0.67 (0.30–1.50)	0.33	0.70 (0.30–1.65)	0.42
Includes Q Fever in Differential for Abortion	0.36 (0.16–0.83)	**0.02**	0.40 (0.17–0.95)	**0.04**
Knows Abortion as Clinical Sign of Q Fever	0.75 (0.34–1.65)	0.47	0.80 (0.35–1.85)	0.61
Knows Q Fever is Spread via Milk or **Aerosols**	0.80 (0.36–1.80)	0.59	0.85 (0.35–2.05)	0.72
Knows that Q Fever is Diagnosed via Blood Tests	0.65 (0.29–1.45)	0.29	0.70 (0.30–1.65)	0.42
Knows that Infected Animals Should Be **Isolated**	0.50 (0.22–1.12)	0.09	0.55 (0.22–1.35)	0.19
Knows that Q Fever Has **Medical Treatment**	0.70 (0.32–1.55)	0.38	0.75 (0.30–1.85)	0.53
Knows a Producer Diagnosed with Q Fever	1.10 (0.50–2.40)	0.81	1.20 (0.50–2.90)	0.68
Knows a Colleague Diagnosed with Q Fever	1.30 (0.60–2.80)	0.51	1.40 (0.60–3.30)	0.43

## Data Availability

The data presented in this study are available on request from the corresponding author.
